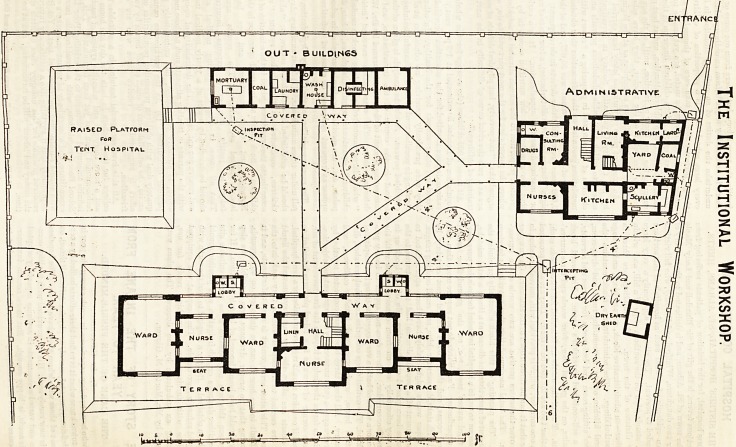# The Sisters' Hospital, St. Alban's

**Published:** 1893-09-02

**Authors:** 


### THE INSTITUTIONAL WORKSHOP.

### THE SISTERS' HOSPITAL, ST. ALBANS. (See next page.)

The Gift of Sir Blundell Maple, M.P.

### HOSPITAL CONSTRUCTION.

### THE SISTERS' HOSPITAL, ST. ALBANS, HERTS.

This hospital, for the isolation of patients suffering from infectious fevers, is the gift of Sir Blundell and Lady Maple to the town of St. Albans, and is dedicated to the memory of their two daughters, Winifred and Dorothy, who fell victims to an epidemic of a kind such as hospitals of this sort are intended to prevent. The site, not apparently a very large one, stands high on the southern slope of a hill, and within easy distance of the town. The buildings are three in number—an administrative block, a ward block, and a range of outbuildings. The administrative block contains the general kitchen offices, a consulting-room and drug-room, nurses' sitting-room and bed-rooms for nurses and servants, also a self-contained caretaker's house. The ward block contains, on the ground floor, four wards and three nurses'-rooms. Two of the wards are intended for three beds each, the other two are for two beds each. They are all approached from one corridor, which is provided with moveable sashes, in order that it can be thrown open in the summer. On an upper floor over the centre portion are two wards and a nurses'-room. The total accommodation thus provided is fourteen beds. The planning of this block effectually precludes the treatment of more than one disease at a time; a fatal mistake, as it seems to us, in a hospital intended for isolation purposes. The third nurses' room, too, on the ground floor would seem superfluous. Four wards do not require more than two nurses' rooms, and these rooms should most certainly be fitted with sinks, dressers for china, and small cooking ranges. The upper wards might safely have been appropriated to the treatment of a second disease had the entrance to the staircase been placed on the other side of the block or in some way effectually cut off from communication with the ground floor corridor. There does not appear to be any provision for a wheeled bath, a very essential thing in a hospital of this kind. The outbuildings comprise an ambulance shed, disinfection house, washhouse, and laundry coalhouse, and mortuary. All these three blocks are connected together by covered ways—a quite unnecessary arrangement and objectionable in so far as they might impede the free movement of air. At one corner of the site is a raised platform intended to be used if occasion should require as a site for a tent hospital. The closets are all on the dry earth system and the liquid slops are carried to a cesspool whence they soak into the ground. This latter arrangement is most unfortunate, and it is difficult to see how it can be defended on any grounds. Assuming that no proper drainage was possible, the slop water should have been disposed of by sub-irrigation. If for any reason this was not possible some more suitable site where either drainage or sub-irrigation was possible should have been obtained. But for a hospital to foul the earth with its liquid refuse is inexcusable. The hospital was equipped complete with furniture and everything ready for use, including an ambulance by the donors. The plans were prepared by Mr. Morton M. Glover.

## Figures and Tables

**Figure f1:**